# The Efficacy of Radiofrequency Ablation Combined with Transcatheter Arterial Chemoembolization for Primary Hepatocellular Carcinoma in a Cohort of 487 Patients

**DOI:** 10.1371/journal.pone.0089081

**Published:** 2014-02-20

**Authors:** Hui Xie, Huaming Wang, Weimin An, Wei Ma, Ruping Qi, Bin Yang, Chunzi Liu, Yuanzhi Gao, Beibei Xu, Wenhong Wang

**Affiliations:** 1 Graduate School of Tianjin Medical University, Tianjin, China; 2 Medical Imaging Center, 302 Hospital of Chinese People’s Liberation Army, Beijing, China; 3 Department of Radiology, Tianjin Union Medical Center, Tianjin, China; West German Cancer Center, Germany

## Abstract

Although diagnostic methods, surgical techniques, and perioperative care have undergone significant advancement over the past decades, the prognosis of primary hepatocellular carcinoma (HCC) remains discouraged because of the high postoperative recurrence rate and high cancer mortality. Radiofrequency ablation (RFA) combined with transcatheter arterial chemoembolization (TACE) is a recently developed means for the treatment of HCC. In this study, we analyzed the efficacy of RFA plus TACE in 487 cases of HCC in our institution. We observed that the 1-, 2-, 3-, 4- and 5-year rates of overall survival rates after RFA and TACE treatment were 97.5% (475/487), 89.4% (277/310), 84.2% (181/215), 80.4% (150/186) and 78.7% (141/177), respectively. We did not find that age or tumor location (the caudate group or non-caudate group) plays a role in this cohort. However, we have identified that tumor recurrent status, the number of tumors, albumin (ALB), prothrombin time (PT) and platelet count (PLT) were significantly associated with poor overall survival in HCC patients receiving RFA combined with TACE. Interestingly, tumor size did not significantly impact overall survival, indicating that RFA combined with TACE for HCC treatment has the same efficiency for different sizes of tumors. Our results provide evidence for the rationale for using combined RFA and TACE in the treatment of primary HCC.

## Introduction

Hepatocellular carcinoma (HCC) is one of the most common human malignancies worldwide and has an estimated diagnosis of 750,000 new cases, with a survival rate of less than 5%, and an average survival of less than a year after diagnosis [1]. In China, HCC is the third leading cause of cancer mortality [2]. Although diagnostic methods, surgical techniques, and perioperative care have undergone significant advancement, the prognosis of HCC patients remains discouraging because of the high postoperative recurrence rate and high cancer mortality. Identifying the optimal therapy to improve outcomes for our HCC patients is therefore crucial for maximizing their long-term survival. Treatment outcomes for HCC patients are affected by multiple variables, such as tumor burden, tumor stage, the Child-Pugh score of liver function reserve and the performance status of the patient [3]. Curative therapy of HCC consists of surgical hepatic resection or liver transplantation (LT). However, liver resection can be done in noncirrhotic patients and a small fraction of cirrhotic patients depending on synthetic dysfunction, degree of portal hypertension, and number and location(s) of tumor(s) [4]. LT is the optimal therapy for patients with HCC, but the shortage of donor organs represents a major problem in applying primary transplantation to many patients. Radiofrequency ablation (RFA) is thought to be the most effective first-line percutaneous ablation technique because of its greater efficacy in terms of local cure compared with ethanol injection [5], [6]. The survival rates for patients who achieved a complete response by RFA are comparable to that of patients treated by hepatic resection [7], [8]. Therefore, RFA has been widely used as a first-line therapy for patients with small HCC who could not receive surgical resection or LT in the recent years [8], [9], [10], [11].

Because HCC is also a type of vascular solid cancer, transcatheter arterial chemoembolization (TACE) and transcatheter arterial embolization (TAE) are widely used for unresectable HCC due to their precisely targeted, minimal invasive, repeatable and well-tolerated features [12], [13], [14], [15]. However, complete necrosis is rarely achieved by TACE or TAE alone due to the incomplete embolization and tumor angiogenesis [16]. In addition, low physiological oxygen levels by TAE may lead to the accumulation of hypoxia-inducible factors and vascular endothelial growth factor (VEGF) and may induce angiogenesis in the residual viable tumor [17].

RFA or TACE has its own limitations. RFA combined with TACE in the treatment of HCC was previously reported and has shown a relatively high complete local response rate compared with TAE or RFA alone [18], [19]. In the current study, we examined the efficacy of RFA combined with TACE for the treatment of HCC in a cohort of 487 patients.

## Materials and Methods

This retrospective study consisted of 487 consecutive patients with solitary HCC, all of whom were treated by RFA combined with TACE in our institution between June 2006 and December 2012. HCC was diagnosed on the basis of standard clinical criteria, imaging criteria and α fetoprotein levels (AFP) according to the American Association for the Study of Liver Diseases practice guidelines on the management of HCC [20], and the diagnosis was confirmed pathologically by needle biopsy. Since World Health Organization defines age >65 years as the elderly [21], this study stratified patients aged 65 years or less as the younger group and those aged >65 years as the elderly group.

### Transcatheter Arterial Chemoembolization (TACE)

Seldinger technology was used to puncture the femoral artery. TACE procedures were performed under radiographic guidelines following the steps that have been described elsewhere [22]. During TACE, a mixture of oxaliplatin, 5-fluorouracil and Doxorubicin was infused into tumor-feeding arteries. Then, super emulsified lipiodol (5–15 ml) was infused into same arteries. A dose of chemical drugs or lipiodol administrated was estimated according to tumor size, number, as well as angiography.

### Radiofrequency Ablation (RFA)

About 2∼4 weeks after finishing TACE treatment, all patients underwent percutaneous RFA using RF2000 radiofrequency system (USA Darrow). Ablation energy and time were determined according to the size and number of tumors in the liver. All tumors were targeted to be ablated with a curative intent in one or two sessions of RFA, with surrounding margins greater than 5 mm. Treatment response was evaluated, and no residual nodular hypervascular region was evidenced by contrast-enhanced computed tomography (CT) after one month post-RFA.

We defined recurrence as the appearance of new lesions with radiologic features typical of HCC, with diagnosis based on at least one imaging method, contrast-enhanced CT or Magnetic Resonance Imaging (MRI). The overall recurrence included tumors occurring at any time or at any site after the patients showed no active lesion at the operation site one month after one or two RFA sessions.

### Biochemical and Serologic Markers

Serum hepatitis B surface antigen (HBsAg) was tested using radioimmunoassay (Abbott Laboratories, North Chicago, IL). Antibody to hepatitis C virus (HCV) was measured by second-generation enzyme immunoassay (Abbott Laboratories, North Chicago, IL). Serum biochemistries, including albumin, bilirubin, alanine aminotransferase, were measured using a systemic multi-auto-analyzer (Technicon SMAC, Technicon Instruments Corp., Tarrytown, NY). Serum AFP level was measured by radioimmunoassay (Serono Diagnostic SA, Coinsin/VD, Switzerland).

### Ethics Statement

Ethical approval was obtained from the Committee of Ethics in Biomedical Research of 302 Hospital of Chinese People’s Liberation Army. Written informed consent was obtained from all participants. The research design was hospital-based and retrospective with all cases being well evaluated.

### Statistical Analysis

GraphPad software was used for data analysis. The data was expressed as mean ± standard deviation. The difference between two groups was determined by a t-test. Pearson x^2^ analysis or Fisher exact test was used to compare categorical variables, whereas the Mann-Whitney U test was used to compare continuous variables. Overall survival rates were estimated by the Kaplan-Meier method and compared using Cox proportional hazards model. P values less than 0.05 were considered statistically significant.

## Results

### Comparison of Clinical Demographic Data

**Figure 1:**
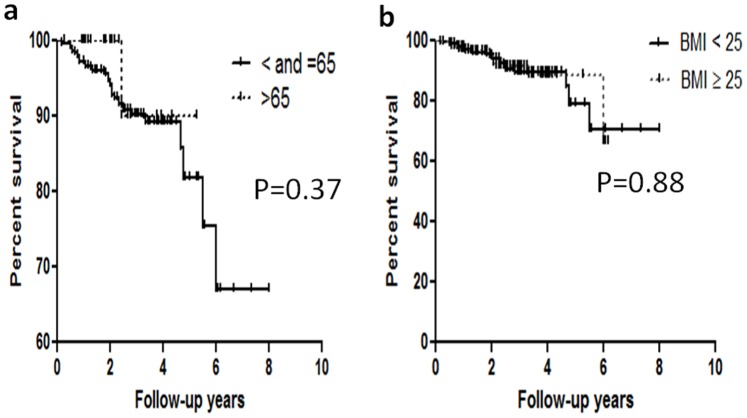
Cumulative overall survival in hepatocellular carcinoma (HCC) patients stratified by age. There was no statistically significant difference in the overall survival rate between young (< = 65 years old) and elderly patients (P = 0.37). The overall survival rates were not significantly correlated with body mass index (BMI) (P = 0.88).

Since previous studies demonstrated that age plays an important role in the outcomes of HCC treatment, we first examined whether or not age also played a role in this cohort. The demographic characteristics of all enrolled patients are shown in **Table 1**. The proportion of sex distribution and background liver diseases (HBV, HCV) of patients was not significantly higher in the younger group than that in the elderly group. There were no significant differences in tumor recurrent status, tumor sizes and numbers between the two groups. In addition, we did not observe significant differences in several biochemical and serologic markers between the two groups. Thus, the overall survival rates were not significantly higher in younger HCC patients than in elderly group (**Figure 1a**). We also determined that the overall survival rates were not significantly correlated with body mass index (BMI) (**Figure 1b**).

**Table 1:** Comparison of the demographic data of younger (Age ≤65 y) and elder (Age >65 y) HCC patients.

Parameter	Younger (*n = *442)	Elder (*n* = 45)	*p*-value
Age (years)	52±7.3	69±3.7	<0.001
Sex (male/female)	371/71	37/8	0.76
Background liver diseases (HBV/HCV/others)	388/30/25	35/6/3	0.75
Naïve/recurrent	423/19	42/3	0.49
Tumor diameter (cm)	2.23±1.19	2.31±1.20	0.67
No. of tumors: >1/1	103/339	11/34	0.86
BMI: ≥25/<25	24.65±3.49	23.66±3.47	0.07
ALT (IU/L)	38.51±26.48	33.20±21.49	0.13
T-Bil (mg/L)	21.49±14.09	18.97±8.64	0.08
ALB (g/dL)	35.83±6.82	35.04±5.63	0.39
PT (%)	13.19±5.59	12.71±1.59	0.18
PLT (x10^4^/µL)	97.56±65.82	95.93±48.13	0.84
AFP	391.79±1752.06	276.69±1123.42	0.55

All data are shown as mean 1 standard deviation. AFP, a-fetoprotein; ALB, albumin; ALT, alanine aminotransferase; HBV, hepatitis B virus; HCV, hepatitis C virus; PLT, platelet; PT, prothrombin time; T-Bil, total bilirubin.

### Factors Associated with Overall Survival After RFA and TACE for HCC

In this study, after RFA and TACE treatment, the 1-, 2-, 3-, 4- and 5-year overall survival rates were 97.5% (475/487), 89.4% (277/310), 84.2% (181/215), 80.4% (150/186) and 78.7% (141/177), respectively. There was a total of 39 cases of death, among which 24 cases died from tumor recurrence, 8 from gastrointestinal bleeding, 3 from liver failure, 2 from hepatorenal syndrome, and 2 from liver portal vein thrombosis.

**Figure 2:**
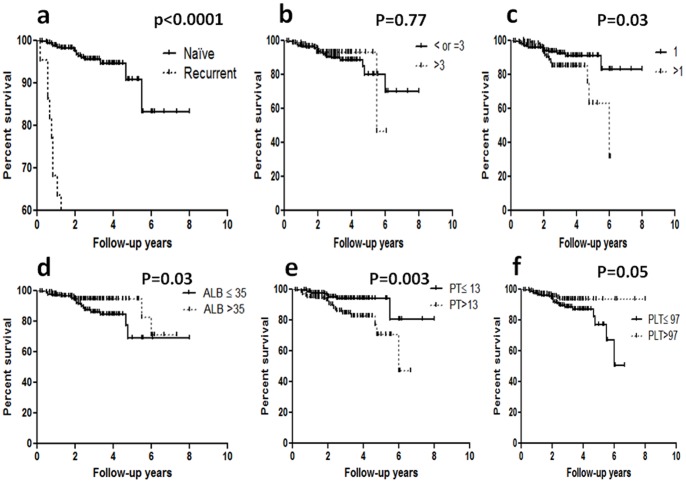
Cumulative overall survival in hepatocellular carcinoma (HCC) patients stratified by naïve or recurrent (a), tumor diameter (cm) (b), number of tumors (c), albumin (ALB) (g/dL) (d), prothrombin time (PT) (e) or platelet count (PLT) (f). The tumor recurrent status, number of tumors, ALB, PT and PLT were significantly associated with poor overall survival after RFA combined with TACE for HCC.

Next, we determined the factors that were associated with poor overall survival after RFA and TACE for HCC by univariate analysis (**Table 2**). We identified that the tumor recurrent status, number of tumors, ALB, PT and PLT were significantly associated with poor overall survival after RFA and TACE for HCC (**Figure 2**). Interestingly, tumor size did not significantly impact the overall survival, indicating that RFA combined with TACE for HCC treatment has the same efficiency for both small and large tumor sizes (**Figure 2a**).

**Table 2:** Factors associated with poor overall survival after RFA and TACE for HCC by univariate analysis.

Variable	Case No.	Hazard Ratio (95% Confidence Interval)	*p*-value
Age: >53/≤54/y/o	257/230	1.56 (0.8295–2.931)	0.16
Sex: male/female	408/79	1.33 (0.5712–3.083)	0.51
Background liver diseases (HBV/HCV/others)	423/36/28	0.33 (0.098–1.109)	0.07
Naïve/recurrent	465/22	1.5×10^−6^(3.01×10^−7^–7.78×10^−6^)	<0.0001
Tumor size: >3 cm/≤3 cm	71/416	1.13 (0.4887–2.600)	0.77
No. of tumors: >1/1	114/373	0.45 (0.2217–0.9328)	0.03
BMI: ≥25/<25	207/280	1.05 (0.5505–1.998)	0.88
ALT (IU/L): >38/≤38	158/329	1.13 (0.5824–2.207)	0.71
T-Bil (mg/L): >21/≤21	178/309	1.01 (0.5230–1.958)	0.97
ALB (g/dL): >35/≤35	260/227	1.98 (1.056–3.754)	0.03
PT (%):>13/≤13	172/315	0.37 (0.1939–0.7106)	0.003
PLT (x10^4^/µL): >97/≤97	182/305	1.87 (0.9764–3.585)	0.05
AFP: >350/≤350	79/408	0.72 (0.2701–1.901)	0.50

All data are shown as mean 1 standard deviation. AFP, a-fetoprotein; ALB, albumin; ALT, alanine aminotransferase; BMI: body mass index; HBV, hepatitis B virus; HCV, hepatitis C virus; PLT, platelet; PT, prothrombin time; T-Bil, total bilirubin.

### Comparison between the Caudate Group and the Non-caudate Group

**Figure 3:**
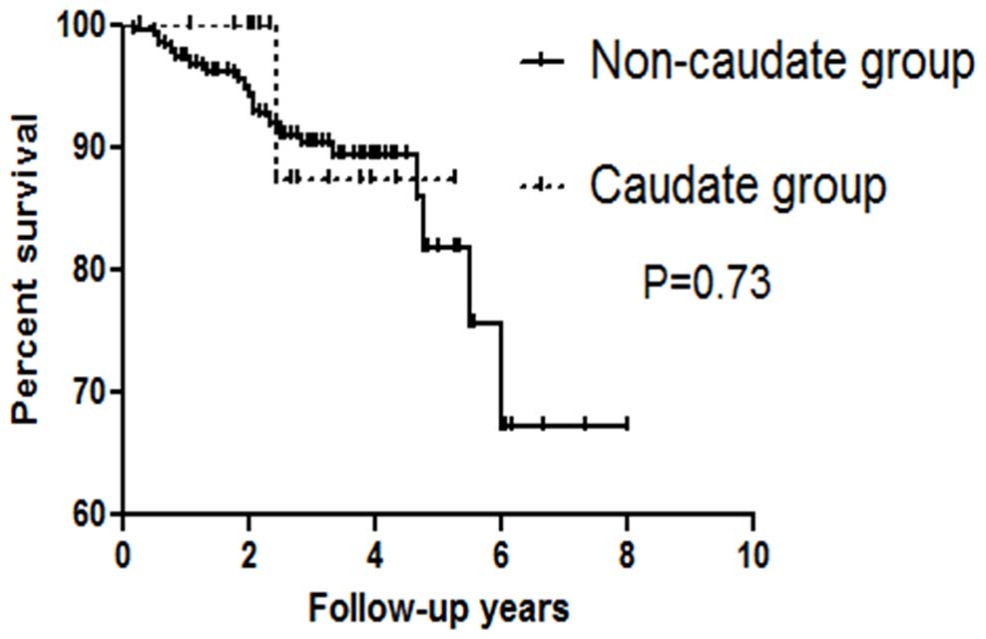
Cumulative overall survival in hepatocellular carcinoma (HCC) patients stratified by the site of tumor. There was no statistically significant difference in the overall survival rate between caudate HCC and non-caudate HCC (P = 0.73).

Finally, we determined whether or not RFA combined with TACE for HCC treatment is as effective for HCC in the caudate lobe as it is for HCC at other sites in the liver (**Table 3**). We did not find any significant differences of several factors between the caudate group and the non-caudate group. As expected, the overall survival rates were not significantly changed between the two groups (**Figure 3**).

**Table 3:** Comparison of baseline factors between the caudate group and the non-caudate group.

Parameter	Caudate group (*n = *19)	Non-caudate group (*n* = 468)	*p*-value
Age (years)	55±8.5	54±8.6	0.48
Sex (male/female)	16/3	392/76	0.94
Background liver diseases (HBV/HCV/others)	16/2/1	407/34/27	0.59
Naïve/recurrent	18/1	447/21	0.79
Tumor diameter (cm)	2.34±1.33	2.47±1.16	0.64
No. of tumors: >1/1	4/15	110/358	0.80
ALT (IU/L)	46.05±33.23	37.69±25.75	0.29
T-Bil (mg/L)	25.07±15.48	21.11±13.62	0.28
ALB (g/dL)	34.16±4.90	35.82±6.78	0.17
PT (%)	13.02±2.07	13.15±5.45	0.79
PLT (x10^4^/µL)	100.12±72.32	97.21±64.09	0.87
AFP	234.27±440.65	387.4±1735.7	0.24

All data are shown as mean 1 standard deviation. AFP, a-fetoprotein; ALB, albumin; ALT, alanine aminotransferase; HBV, hepatitis B virus; HCV, hepatitis C virus; PLT, platelet; PT, prothrombin time; T-Bil, total bilirubin.

## Discussion

HCC is one of the most common malignant tumors in China, with the incidence rate up to 80 per million of the populations. More than 80% patients have simultaneous liver cirrhosis, which leads to an actual rate of resection less than 30%, and the 5 year recurrence rate after resection reaches above 70% [23], [24], [25]. Therefore, RFA is generally considered as an alternative treatment to partial hepatectomy for early HCC, especially for patients with impaired liver function and when liver transplantation is not indicated. RFA is also recommended as a first-line treatment for early HCC [26], [27], [28]. TACE or TAE is another minimally invasive option that may achieve the pertinent balance in successful tumor eradication and maximal preservation of liver functions [29], [30]. Theoretically, TACE that combines the effects of transcatheter arterial chemotherapy and TAE should be more effective than either alone, a meta-analysis of TACE *versus* TAE alone has demonstrated no survival difference [31]. RFA combined with TAE in the treatment of HCC was seldom reported, however, the synergy between RFA and TACE has been well described [18], [32]. Occlusion of hepatic arterial flow by embolization reduces the cooling effects of hepatic blood flow on thermal coagulation. In addition, iodized oil and gelatin sponge particles used in TACE fill the peripheral portal vein around the tumor by going through multiple arterioportal communications, thus reducing the portal venous flow [22], [33]. TACE or TAE, being a regional treatment, can target undetected satellite lesions surrounding the zone of RFA-induced necrosis. Therefore, RFA combined with TAE or TACE is increasingly used in the treatment of HCC in patients with well-compensated liver diseases.

In this study, we determined that the 1-, 2-, 3-, 4- and 5-year overall survival rates after RFA and TACE treatment were 97.5% (475/487), 89.4% (277/310), 84.2% (181/215), 80.4% (150/186) and 78.7% (141/177), respectively. Shibata et al [34] reported the overall survival rates of the 1-, 2-, 3- and 4-year, from combined TACE and RFA (n = 46) compared with RFA alone (n = 43) in patients with Child’s A or B cirrhosis and resectable HCC with ≤3 nodules smaller than 3 cm. The overall survival rates were 100, 100, 84.8 and 72.7%, respectively, in the combined treatment group and 100, 88.8, 84.5 and 74.0%, respectively, in the RFA group (p = 0.515). Thus, the authors considered that combined RFA plus TACE and RFA alone have equivalent effectiveness for the treatment of small-sized (≤3 cm) HCCs, and they thought the combination of treatments may not be necessary. Morimoto et al. [28] reported the 3-year survival rates of the patients in the RFA and TACE-RFA groups were 80 and 93%, respectively (p = 0.369) from a midterm outcomes of a randomized controlled trial comparing the efficacy of TACE combined with RFA with RFA alone for the treatment of intermediate-sized HCC. Recently, Peng et al [35] reported that the 1-, 3-, and 4-year overall survivals for the TACE-RFA group in the treatment of HCC were 92.6%, 66.6%, and 61.8%, respectively, whereas in the RFA group, they were 85.3%, 59%, and 45.0%, respectively. They concluded that TACE-RFA was superior to RFA alone in improving survival for patients with HCC less than 7 cm. In our study, tumor size did not significantly impact the overall survival indicating that RFA combined with TACE for HCC treatment is the same effective for big sized tumors (>3 cm) as for small sized tumors (≤3 cm). Although the role of combined RFA with TAE or TACE in the treatment of HCC has not completely determined, the published results and our current study provide evidence for the rationale for using combined RFA with TAE or TACE in the future treatment of primary HCC. It should be noted that yttrium-90 microspheres as radioactive particles are also increasingly employed for treating patients with unresectable HCC, which is called radioembolization [36], [37]. Further studies will be needed to identify which option is the best for treating patients with unresectable HCC.

Since previous studies reported younger HCC patients have better overall survival and lower recurrence rate after RFA compared with elder patients [9], we examined whether or not age also plays a role in this cohort. We found the overall survival rates were not significantly higher in younger than in elderly HCC patients. Since previous report showed that the 4-year cumulative local recurrence rate after RFA in the caudate group and the non-caudate group was 22.3% and 4.5%, respectively (*P*<0.001) [10], we also determined whether or not RFA combined with TACE for HCC treatment is as effective for HCC in the caudate lobe as it is for HCC at other sites in the liver. We did not find any significant differences between the caudate group and the non-caudate group and the overall survival rates were not significantly changed between the two groups.

In conclusion, this study identified that tumor recurrent status, number of tumors, ALB, PT and PLT were significantly associated with poor overall survival after RFA and TACE for HCC. We did not observe any significant difference in the overall survival between younger and elderly patients, or patients with caudate and non-caudate HCC. We also found that tumor size or patient age does not affect the overall survival. This is different from another study reporting that the tumor size and number were significant independent factors affecting recurrence-free survival [38]. In order to achieve better efficacy for treating patients with unresectable HCC, Further studies will be needed to identify the prognostic factors that could potentially affect the outcome of this combination therapy.
